# The Effect of Patient Characteristics and Sleep Quality on Visual Field Performance Reliability

**DOI:** 10.1155/2018/2731260

**Published:** 2018-02-12

**Authors:** Swarup S. Swaminathan, Matthew B. Greenberg, Elizabeth A. Vanner, Kara M. Cavuoto, Sarah R. Wellik, Ta Chen Chang

**Affiliations:** ^1^Bascom Palmer Eye Institute, Miami, FL, USA; ^2^University of Miami Miller School of Medicine, Miami, FL, USA

## Abstract

**Purpose:**

To investigate the association of automated visual field (VF) reliability indices (false positive [FP], false negative [FN], and fixation loss [FL]) and sleep quality, VF experience, and age.

**Methods:**

Prospective, cross-sectional study. Adult patients (age ≥ 18 years) completing automated VF testing were invited to participate. Baseline participant characteristics were obtained, and all participants were asked to complete the Pittsburgh Sleep Quality Index (PSQI) questionnaire. Nonparametric Spearman correlations and logistical regression models were performed.

**Results:**

63 patients were enrolled. Lower PSQI score was correlated with higher percentage (%) FL in the right eye (*p* = 0.03). Fewer prior VF was significantly correlated with higher %FP in the right eye (*p* = 0.008). Older age was significantly correlated with higher %FN in the left eye (*p* = 0.01). Greater mean deviation (MD) and pattern standard deviation (PSD) were strongly correlated with higher %FN in the right (*p* = 0.02 and 0.002, resp.) and left eyes (*p* = 0.01 and 0.02, resp.).

**Conclusion:**

In this prospective, cross-sectional study, worse MD and PSD are strongly correlated with increased FN in both eyes. Increased FN in the left eye associated with older age might be attributable to test fatigue. Worse sleep quality is associated with decreased FL in the right eye.

## 1. Introduction

Automated visual field (VF) examination is an important ancillary test in the care of ophthalmic patients, with over 3 million performed annually [1]. VF reliability indices (fixation loss [FL], false positive [FP], and false negative [FN]) are used to monitor test precision and reliability [2]. Whereas reliable tests yield valuable clinical information, unreliable tests are not clinically useful and squander significant resources and time. Thus, identifying high-risk patient characteristics of poor VF performance may allow more judicious allocation of time and resources in patient management. Prior publications had reported FN as an important metric in the evaluation of glaucoma [3]. However, a recent publication evaluating over 10,000 VFs demonstrated that among all reliability indices, FP had the greatest impact on VF reliability [4]. Both FN and FP can affect mean deviation (MD), with FP increasing MD and FN decreasing MD; the greater the magnitude of FN or FP, the greater the effect on MD [5]. Reliability indices clearly have a significant role not only on the quality of the study, but also on the assessment of glaucomatous severity.

In glaucoma patients, older age and more severe VF defects have been associated with poor VF reliability [6], while acute sleep deprivation was associated with a significant decrease in VF reliability, with sensitivity to these stressors increasing with age [7]. Furthermore, sleep loss has been linked to increased reaction time and poor task performance [8, 9], which may contribute to poor performance on automated VF examinations. However, no prior studies evaluating the impact of sleep quality on VF performance with the use of a validated questionnaire were found.

The Pittsburgh Sleep Quality Index (PSQI) is a validated questionnaire instrument in sleep quality assessment that has demonstrated high degrees of test-retest reliability and validity in the diagnosis of sleep disorders [10]. We hypothesize that, in addition to patient characteristics and the extent of visual field damage, sleep quality as assessed by PSQI may be associated with VF reliability.

## 2. Methods

A prospective, cross-sectional study of consecutive patients was conducted between December 1, 2016, and February 1, 2017. Approval was obtained from the Institutional Review Board (IRB) of the University of Miami Miller School of Medicine, and the study complied with the tenets of the Declaration of Helsinki and was HIPAA compliant. Patients included were adults (age ≥ 18 years) who were scheduled for a 24-2 Humphrey VF examination (Swedish Interactive Threshold Algorithm standard 24-2 strategy, Humphrey Field Analyzer 750 II-I, Carl Zeiss Meditec Inc., Dublin, CA) of both the right eye (OD) and the left eye (OS) at the Bascom Palmer Eye Institute. Other inclusion criteria included fluency in English or Spanish in order to complete the PSQI questionnaire. Each eligible patient participated only once, even if multiple VF exams were performed during the study period. All eligible patients were invited to participate.

After obtaining informed consent, patient characteristics were recorded, and the patient underwent the scheduled VF examination and completed the PSQI questionnaire. The questionnaire had to be completed entirely and according to the instructions in order to properly calculate a PSQI score; incomplete questionnaires were excluded. The VF and survey data were aggregated in a de-identified fashion. The PSQI score was calculated based on responses to the questions as per the questionnaire protocol [11]. Spearman correlations were used to assess the association between PSQI scores and age/visual field characteristic. Univariate and multivariate regression models were performed to assess potential confounding among variables. Paired *t*-tests were completed using SAS statistical software (SAS, Cary, NC.)

## 3. Results

A total of 63 patients were included in the study, with an average age of 65.8 ± 14.8 years. Overall, the average VF defects were mild (−4 ± 6.9 dB), and the patients have completed an average of 2.7 ± 3.3 prior VF examinations. The average PSQI score was 6.17 ± 3.73 with 52% scoring more than 5 points, reflecting poor sleep quality. Patient characteristics are summarized in Table 1.

Correlating PSQI scores with FP, FN, and FL in both eyes, the only significant association was with the percentage (%) of FL OD (*r* = −0.28, *p* = 0.03; Table 2). This was a negative correlation, with high PSQI scores (worse sleep quality) correlating with decreased %FL OD. All other reliability indices were not significantly correlated with the PSQI scores.

**Figure 1:**
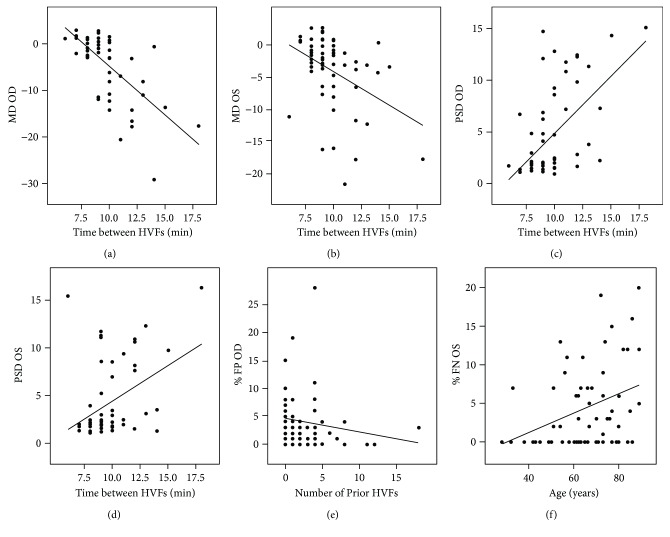
Scatterplots demonstrating the correlations between time between visual fields (VFs) and (a) mean deviation (MD) of the right eye, (b) MD of the left eye, (c) pattern standard deviation (PSD) of the right eye, and (d) PSD of the left eye. Of note, one outlier with time between VFs of 34 minutes was removed from graph (a)–(d). Scatterplots demonstrating correlation between (e) number of prior VFs and false positive percentage of the right eye and (f) age and false negative percentage of the left eye.

Number of prior VF, extent of VF damage, and age were significantly correlated with VF reliability indices (Figure 1). Fewer prior VF was significantly correlated with higher %FP OD (*r* = −0.34, *p* = 0.008), while older age was significantly associated with higher %FN OS (*r* = 0.33, *p* = 0.01). More severe disease was strongly associated with FN, as greater magnitude of MD and pattern standard deviation (PSD) was strongly associated with higher %FN OD (*r* = −0.38, *p* = 0.002 and *r* = 0.38, *p* = 0.002, resp.) and OS (*r* = −0.31, *p* = 0.01 and *r* = 0.30, *p* = 0.02, resp.). Of note, foveal sensitivity did not significantly differ between the two eyes (33.5 ± 7.4 dB OD, 34.5 ± 5.6 dB OS; *p* = 0.40).

Patients who took a greater amount of time between the two eyes usually had a greater magnitude of MD and PSD OD (*r* = −0.61, *p* < 0.0001 and *r* = 0.56, *p* < 0.0001, resp.) and OS (*r* = −0.40, *p* = 0.001 and *r* = 0.28, *p* = 0.03, resp.; Figure 1), suggesting that patients with more severe VF damage were more likely to require more time between the examinations of the right and left eyes. Notably, one significant outlier with a time between VFs of 34 minutes was removed from the analysis. Older patients also usually required more time between exams (*r* = 0.27, *p* = 0.04). There was no correlation between reliability indices and the identity of any given visual field technician (data not shown).

## 4. Discussion

Poor performances on automated VF examination have significant financial and logistical implications. An analysis of Medicare data shows that over 3 million VF examinations are completed yearly, costing approximately $200–$300 million [1]. Understanding potential contributors to poor VF performance would allow better resource allocations.

Although anecdotal evidence suggested that sleep quality may affect VF performance, our prospective study did not demonstrate any clinically significant association between sleep quality as assessed by the PSQI and VF reliability indices. The relationship between PSQI score and %FL OD was statistically significant, but given the lack of any other reliability index associations with PSQI score and the inverted nature of the correlation coefficient, this association may have been due to chance. While statistically significant, we do not believe that this result is clinically significant. It is important to note here the main limitation of the PSQI, which is that it is self-reported. In addition, it is unique in its characterization of an activity during which the individual is unconscious, therefore making self-assessment somewhat challenging, although it is a well-studied and verified metric. The survey does include questions regarding the opinions of a cohabiting partner, but these are not included in the calculation of the PSQI score.

In our cohort, fewer prior VF examinations were correlated with increased %FP OD, perhaps due to test-related anxiety [12]. By convention, our institution tests the right eye first, and it is not surprising that increased FP is no longer noted in the subsequently tested left eye, as patients would have received feedback to improve reliability by the time the test is performed on the left eye. However, when testing the second (left) eye, older patients have higher %FN, possibly due to inattention and/or fatigue. We found worse MD and PSD to be strongly correlated with higher %FN in both eyes, which is consistent with prior studies [6, 13]. The mechanism of this phenomenon is presumably “response saturation,” such that decreased ganglion cell density may result in longer refractory period and a failure to respond to a repeated stimulus [14]. Of note, foveal sensitivities were similar between the two eyes as noted above, suggesting that there was unlikely to be confounding by visual acuity. We were unable to collect actual visual acuity data due to limitations of our IRB-approved protocol.

Patients with more severe VF damage required more time between testing OD and OS. While the cause of this phenomenon remains uncertain, we suspect that perhaps the VF technicians felt greater need to repeat testing instructions between eyes given the propensity for these patients to have higher %FN. The patients may have requested a longer rest time between eyes due to physiologic adaptation of a higher refractory period. Further studies are needed to elucidate the nature of this phenomenon. Of note, the results of univariate and multivariate regression models indicated that there was no confounding among the variables (in Table 1) for which we assessed Spearman correlations with PSQI scores.

Our study has a few limitations. PSQI is a self-reported questionnaire, as previously mentioned, and may not be sufficiently sensitive in detecting sleep problems that may affect VF performance. While consecutive eligible patients were invited to participate, we cannot exclude the possibility of selection bias such that only those patients who were relatively well rested chose to complete a lengthy questionnaire in addition to performing automated VF examinations. It is important to note that patients who did not complete the PSQI questionnaire in its entirety, or did not follow the questionnaire instructions, were excluded, which may have biased against poorly rested patients. The VF examination instructions from the technicians were not scripted nor scheduled, and it is plausible that patients who would have otherwise performed poorly received additional coaching and/or had their tests started over, which may have blunted the study effect. Lastly, we cannot exclude the possibility that the sample size is simply not sufficiently large to detect a subtle effect, although the effect is not likely to be sufficiently robust as to be clinically relevant.

In summary, VF reliability indices were not affected by sleep quality as assessed by PSQI scores, but do appear to be affected by other patient characteristics, which can impact the overall VF testing experience. Future studies may involve finding strategies to improve reliability in patients with a history of poor performances. In addition, we may consider randomizing the laterality when initiating the visual field to evaluate the fatigue phenomenon further. Implications of this study include the consideration of additional coaching prior to starting the VF testing for those who are at highest risk for poor VF performance—older patients, those with more severe VF damage, and those with little prior VF experience. Additional assistance could help avoid costly, minimally useful visual field testing.

## Figures and Tables

**Table 1:** Patient characteristics.

Patient characteristics (*n* = 63)	
Age (years)	65.8 ± 14.8
% Male	45%
Number of prior VF	2.7 ± 3.3
MD OD (dB)	−4.36 ± 6.9
PSD OD (dB)	4.79 ± 4.33
Foveal sensitivity OD (dB)	33.5 ± 7.4
MD OS (dB)	−3.94 ± 6.1
PSD OS (dB)	4.26 ± 4.0
Foveal sensitivity OS (dB)	34.5 ± 5.6
PSQI score	6.17 ± 3.73

SD = standard deviation; VF = visual field; MD = mean deviation; OD = right eye; PSD = pattern standard deviation; OS = left eye; PSQI = Pittsburgh Sleep Quality Index.

**Table 2:** Spearman correlations between visual field characteristics and PSQI scores.

	PSQI score	
	*r*	*p* value
%FP OD	−0.106	0.42
%FN OD	−0.087	0.51
%FL OD	−0.276	0.03^∗^
MD OD	−0.003	0.97
PSD OD	0.062	0.64
%FP OS	−0.208	0.11
%FN OS	0.133	0.31
%FL OS	−0.023	0.86
MD OS	−0.065	0.62
PSD OS	−0.005	0.97
Time between VF (min)	−0.002	0.99
Age	−0.023	0.86
Number of prior VF	−0.052	0.69

PSQI = Pittsburgh Sleep Quality Index; FP = false positive; OD = right eye; FN = false negative; FL = fixation loss; MD = mean deviation; PSD = pattern standard deviation; OS = left eye; VF = visual field.
